# The respiratory control of carbon dioxide in children and adolescents referred for treatment of psychogenic non-epileptic seizures

**DOI:** 10.1007/s00787-017-0976-0

**Published:** 2017-03-24

**Authors:** Kasia Kozlowska, Reena Rampersad, Catherine Cruz, Ubaid Shah, Catherine Chudleigh, Samantha Soe, Deepak Gill, Stephen Scher, Pascal Carrive

**Affiliations:** 1 Psychological Medicine, The Children’s Hospital at Westmead, Locked Bag 4001, Westmead, NSW 2145 Australia; 2 Brain Dynamics Centre, Westmead Institute for Medical Research, Westmead, NSW Australia; 3 University of Sydney Medical School, Sydney, NSW Australia; 4 Lady Cilento Children’s Hospital, Brisbane, QLD Australia; 5 Department of Psychiatry, Harvard Medical School, Boston, MA USA; 6 McLean Hospital, Belmont, MA USA; 7 Department of Anatomy, School of Medical Sciences, University of New South Wales, Sydney, NSW 2052 Australia

**Keywords:** Non-epileptic seizures, Functional neurological symptom disorder, Conversion disorder, Dissociative convulsions, Hyperventilation

## Abstract

Psychogenic non-epileptic seizures (PNES) are a common problem in paediatric neurology and psychiatry that can best be understood as atypical responses to threat. Threats activate the body for action by mediating increases in arousal, respiration, and motor readiness. In previous studies, a range of cardiac, endocrine, brain-based, attention-bias, and behavioral measures have been used to demonstrate increases in arousal, vigilance, and motor readiness in patients with PNES. The current study uses respiratory measures to assess both the motor readiness of the respiratory system and the respiratory regulation of CO_2_. Baseline respiratory rates during clinical assessment and arterial CO_2_ levels during the hyperventilation component of routine video electroencephalogram were documented in 60 children and adolescents referred for treatment of PNES and in 50 controls. Patients showed elevated baseline respiratory rates [*t*(78) = 3.34, *p* = .001], with 36/52 (69%) of patients [vs. 11/28 (39%) controls] falling above the 75th percentile (*χ* ^2^ = 6.7343; *df* = 1; *p* = .009). Twenty-eight (47%) of patients [vs. 4/50 (8%) controls] showed a skewed hyperventilation-challenge profile—baseline PCO_2_ <36 mmHg, a trough PCO_2_ ≤ 20 mmHg, or a final PCO_2_ <36 mmHg after 15 min of recovery—signaling difficulties with CO_2_ regulation (*χ* ^2^ = 19.77; *df* = 1; *p* < .001). Children and adolescents with PNES present in a state of readiness-for-action characterized by high arousal coupled with activation of the respiratory motor system, increases in ventilation, and a hyperventilation-challenge profile shifted downward from homeostatic range. Breathing interventions that target arousal, decrease respiratory rate, and normalize ventilation and arterial CO_2_ may help patients shift brain–body state and avert PNES episodes.

**Electronic supplementary material:**

The online version of this article (doi:10.1007/s00787-017-0976-0) contains supplementary material, which is available to authorized users.

## Introduction

Psychogenic non-epileptic seizures (PNES) is a nonspecific, umbrella category referring to non-epileptic seizures that are of presumed emotional or psychological origin, are not explained by neurological disease, and are thought to reflect abnormal central nervous system functioning [1, 2]. PNES are diagnosed following a comprehensive evaluation involving a detailed history and negative finding of ictal electrical activity on electroencephalogram [either video EEG (vEEG) or EEG telemetry] [3]. A limitation of the diagnosis is that the neurophysiological mechanisms underlying the seizures remain unexplained, whereas the current move within neurology and psychiatry is to identify the biological mechanisms that underpin different subtypes of PNES [4–7].

A growing number of studies have examined the neurophysiological characteristics of patients with PNES. Key findings include: (1) increased baseline arousal indexed by elevated cortisol, elevated heart rate (HR), decreased heart rate variability (HRV) [8–10]^1^; (2) exaggerated neurophysiological responses—lower HRV recovery, increased cortisol release, and increased amplitudes of event-related potentials (ERPs)—to psychological or physiological stress stimuli [8, 14, 15]; (3) increased vigilance, and motor readiness to respond, to emotional signals [8, 11]; (4) changes in connectivity in resting-state brain networks, with alterations in brain circuits mediating emotion regulation and arousal, cognitive control, self-referential processing, and motor planning and coordination [5]; (5) changes in EEG synchrony, both within cortical brain systems and between cortical and subcortical brain systems [16–19]; (6) arousal-related impairments in prefrontal cortex function [12, 14]; and (7) increased avoidance tendencies to social-threat cues [20]. Taken together, these findings suggest that PNES may emerge in the context of an upregulated stress system and extreme reactivity to psychological or physiological threat stimuli.

Threat stimuli prepare the body for action by mediating increases in arousal, respiration, and motor readiness. Increases in ventilation—indexed by breathing rate and tidal volume—occur alongside a multitude of other changes. When the breathing exceeds metabolic demands—termed hyperventilation (HV)—there is a depletion of CO_2_ stores, a respiratory alkalosis (high pH), and biphasic brain response [see Supplemental Text Box 1 (online) for a summary of the neurophysiology of hyperventilation]. An initial excitatory phase is marked by an increase in cortical excitability in widely distributed networks [21, 22]. A subsequent hypoxic phase, mediated by cerebral artery constriction, involves a decrease in cortical function [23–25]. This hypoxic phase is marked by central symptoms, including: impaired mental performance/gaps in memory; dizziness and lightheadedness; visual phenomena (blurred, clouded, or tunnel vision; flashing lights; or complete blackout); and changes in level of consciousness ranging from disorientation/staring to a complete loss of consciousness (syncope) [23, 26, 27]. The degree of hypoxia is determined by, and proportional to, individual variations in cerebral artery constriction [28, 29]. Because the developing brain is more sensitive to the central effects of HV than the adult brain [25, 30], children/adolescents are likely to be more vulnerable than adults to HV-induced changes in brain function.

In the paediatric literature, PNES secondary to HV have been described in case reports since the 1960s [31–45], but data from larger cohort studies are sparse. In two Indian studies examining the clinical characteristics of youth referred to psychological services for functional neurological symptom disorder, a quarter of the youth presenting with PNES experienced HV [6/21 (29%) in one study; 6/25 (24%) in the other], with or without fainting spells [40, 41]. A Hungarian study of PNES during vEEG documented HV in 14/75 (18.7%) of child/adolescent patients [46]. In adult studies, approximately 70–83% of patients report multiple panic-like symptoms—presumably accompanied by increased respiratory rate—during PNES [47, 48]. Taken together, the above-described studies suggest that panic-like symptoms or hyperventilation occur in association with some PNES, with some indication that hyperventilation may play a causal role.

The current study used respiratory measures to assess the motor readiness of the respiratory system and the respiratory regulation of CO_2_ in children/adolescents referred for treatment of PNES and in controls. We hypothesized that, compared to controls, patients with PNES would show elevated baseline respiratory rates at clinical assessment and aberrant patterns of PCO_2_ regulation during the hyperventilation component of the vEEG, including (1) lower baseline resting PCO_2_ levels, (2) faster drops in PCO_2_, (3) lower PCO_2_ levels following 5 min of routine hyperventilation during the vEEG procedure, and (4) less efficient recoveries back to a homeostatic norm (≥36 mmHg) following hyperventilation. We also expected that compared to healthy controls, patients with PNES would show elevated baseline HR, in line with previous findings [10].

## Methods

### Participants

In 2011, in a joint Neurology–Psychological Medicine project with children/adolescents being assessed for PNES—The PNES Hyperventilation Study—we added both a percutaneous probe to the vEEG to measure PCO_2_ and a biofeedback device (MyCalmBeat) to measure baseline respiratory rate and implement breath-control interventions [10]. In the present study we report on 60 children/adolescents, recruited from April 2011 to March 2016, who were diagnosed and treated for PNES. Four children/adolescents were excluded because PCO_2_ data were not collected, and four because PCO_2_ data were inadequate (technical difficulties or child’s lack of cooperation).

Potential controls were excluded from the study if they had a past history of PNES, suffered from, or had a past history of, panic attacks or hyperventilation, or experienced an epileptic seizure during the EEG and were unable to follow instructions.

The study was approved by the Sydney Children’s Hospital Network Ethics Committee. Participants and their legal guardians provided written informed consent in accordance with the National Health and Medical Research Council guidelines.

### Procedure

#### Video EEG

Children/adolescents with PNES underwent a routine vEEG as part of their neurological assessments. Psychiatry controls underwent the same procedure as part of their inpatient medical workups, epilepsy controls as part of their epilepsy treatment, and healthy controls as volunteer participants for the study. The procedure (35 min) involved: an eyes-open condition (3 min); an eyes-closed condition (2 min); photic stimulation (3 min); a 5-min break; and the HV challenge itself (5 min of HV and a 15-min recovery period). The full 10–20 electrode placement plus a single-channel electrocardiogram was used. A pulse oximeter, placed on the second finger, recorded the HR and peripheral oxygen saturation, and a percutaneous PCO_2_ monitor attached to the inner forearm measured the blood CO_2_ level throughout the procedure. The baseline resting PCO_2_ level was collected immediately before the commencement of HV (the zero value in the 20-min HV time series). High-amplitude generalized slowing on the EEG was an indicator of good physiological response to hyperventilation. The EEG scientist also used a pinwheel as a visual indicator, to encourage compliance and increase effort. All vEEG data were reviewed by a neurologist.

#### MyCalmBeat biofeedback assessment

All patients diagnosed with PNES attended a Psychological Medicine evaluation—a family assessment [49] and an individual assessment, where MyCalmBeat was used to document baseline respiratory rate and to assess the children/adolescents’ ability to slow down their breathing rates to upregulate vagal tone. Healthy controls completed the vEEG procedure and MyCalmBeat on the same day.

### Data analysis

Table 1 lists the measures used for data analysis. A PCO_2_ value of <36 mmHg was considered to indicate hypocapnia [50, 51]. The term *skewed HV*-*challenge profile* was used to describe a HV-PCO_2_ profile that was shifted down and that was defined by baseline PCO_2_ < 36 mmHg, trough PCO_2_ ≤ 20 mmHg, or final PCO_2_ < 36 mmHg after 15 min of recovery. The PCO_2_ cutoff of ≤20 mmHg was used for the trough-PCO_2_ reading that defined a skewed HV-challenge profile because central symptoms are more likely at ≤PCO_2_ 20 mmHg (range 14–29 mmHg) [52]. A respiratory rate above the 75th percentile—≥19 breaths per minute for children 8–11 years and ≥21 breaths per minute for adolescents 12–15 years—was considered to be elevated. A HR above the 75th percentile—≥100 bpm for children 8–12 years and ≥90 bpm for children >12 years—was considered to be elevated. These values for respiratory rate and HR were derived from normative reference ranges [53].

**Table 1:** Description of measures from the hyperventilation (HV) challenge and MyCalmBeat assessments

Measure	Description	Units
Baseline HR and respiratory rate		
Clinical baseline breathing rate	The baseline breathing rate collected during the MyCalmBeat assessment	Breath per minute
Baseline HR	The baseline HR value from the start of the HV challenge (zero value on Fig. 1)	bpm
Hyperventilation challenge PCO_2_ measures		
Baseline PCO_2_	The baseline PCO_2_ value from the start of HV challenge (zero value on Fig. 1)	mmHg
∆PCO_2_	Change in PCO_2_—the size of the PCO_2_ drop—calculated as HV baseline PCO_2_ minus lowest PCO_2_ level during HV challenge	mmHg
Trough PCO_2_	Lowest PCO_2_ value during HV challenge	mmHg
Time to trough PCO_2_	Time from HV baseline PCO_2_ (zero value) to trough-PCO_2_ value	minutes
Rate of PCO_2_ drop	Rate of drop from HV baseline PCO_2_ (zero value) to trough-PCO_2_ value	mmHg/min
Rate of PCO_2_ recovery	Rate of elevation of PCO_2_ from trough-PCO_2_ value during recovery period was calculated from trough-PCO_2_ value during HV challenge to first value in the homeostatic range (≥36 mmHg) (if homeostasis was attained) trough-PCO_2_ value during HV challenge to final PCO_2_ value after termination of HV challenge and 15 min of recovery (if homeostasis was not attained)	mmHg/min
Final PCO_2_	Final PCO_2_ reading after termination of HV challenge and 15 min of recovery	mmHg
Other terms used		
Skewed HV profile	A HV profile that was shifted down and defined by baseline PCO_2_ < 36 mmHg, trough-PCO_2_ ≤ 20 mmHg, or final PCO_2_ < 36 mmHg baseline	

A general linear model for multiple analyses was used to examine the difference in the HV-challenge curve between the PNES group and controls. Chi-square analyses and independent *t* tests were used to calculate differences between the PNES group and the merged control group (see below) on categorical and continuous variables, respectively. For respiratory measures, all control children/adolescents were combined into one group because the three control groups showed no significant differences on any measures (see Table 2). For HR measures, the PNES group was compared only to healthy controls because baseline HR was elevated in both clinical control groups (see Table 2).

**Table 2:** Study measure comparisons: healthy controls vs. psychiatry controls vs. epilepsy controls

Measure	Mean value for psychiatry controls (*n* = 25)	Mean value for healthy controls (*n* = 17)	Mean value for epilepsy controls (*n* = 8)	*t* (*p* value) psychiatry vs. healthy controls epilepsy vs. healthy controls
Baseline HR and respiratory rate				
Baseline HR (bpm)	87.16	77.24	86.50	−2.412 (0.021) −1.870 (0.074)
Clinical baseline breathing rate (breaths per minute)	18.00 range (8–24)	19.71 range (8–31)	Missing data	1.628 (0.116) –
Hyperventilation challenge PCO_2_ measures				
Baseline PCO_2_ (mmHg)	41.20	41.06	41.13	−0.130 (0.897) −0.042 (0.967)
∆PCO_2_ (mmHg)	13.88	15.47	17.00	1.397 (0.170) −0.944 (0.355)
Trough-PCO_2_ (mmHg)	27.32	25.59	25.00	−1.435 (0.159) 0.485 (0.632)
Time to trough PCO_2_ (min)	5.16	5.53	4.88	1.469 (0.150) 1.674 (0.108)
Rate of PCO_2_ drop (mmHg/min)	2.76	2.89	2.83	0.451 (0.654) −1.905 (*p* = .069)
Recovery measures				
Rate of PCO_2_ recovery (mmHg/min)	4.00	5.56	3.63	1.482 (0.153) 1.302 (0.206)
Final PCO_2_ (mmHg)	41.26	43.65	44.13	−1.012 (0.318) −1.641 (0.114)

## Results

### Clinical characteristics of participants assessed for PNES

The clinical cohort consisted of 60 children/adolescents—42 girls and 18 boys, aged 8–17 years [mean = 13.45; standard deviation (SD) = 2.61]—undergoing vEEG for assessment of PNES. Of the 60 patients, 7 presented with a clinical diagnosis of HV at referral.

The time from onset of PNES ranged from one day to 48 months (median = 2 months). In 28 cases (47%) the PNES presented alongside other functional neurological symptoms; in 10 cases (17%) the PNES presented alongside chronic pain; and in 22 cases (36.66%) the PNES were the primary presenting symptom. PNES semiology included the following: movements (rhythmic, thrashing/kicking, flexion/extension) (*n* = 15); syncopal-like events alone (*n* = 11); visual blackout, loss of vision, or changes in consciousness associated with head dropping (*n* = 8); prolonged periods of unresponsiveness (*n* = 2); sensory experiences (*n* = 2); changes in responsiveness followed by amnesia lasting days or weeks (loss of memory of self or parents) (*n* = 2); staring episodes (*n* = 1); both movements and syncopal-like events (*n* = 17); and movements, syncopal-like events, and long periods of unresponsiveness (*n* = 2).

A third of children/adolescents (20/60; 33.33%) suffered from comorbid neurological conditions (see Table 3). All families reported antecedent stressors (range, 1–12; mean = 4.63; median = 4): an illness event (accident, infection, or diagnosis or relapse of a chronic illness) (30/60; 50%); family conflict (26/60; 43%); maternal mental illness (typically anxiety or depression) (26/60; 43%); being bullied (23/60; 38%); loss due to separation (21/60; 35%); paternal mental illness (16/60; 27%); loss due to death (13/60; 22%); or exposure to domestic violence (12/60; 20%). Maltreatment was less common: sexual abuse (8/60; 13%); physical abuse (7/60; 12%); or neglect (7/60; 12%). Comorbid motor-sensory conversion symptoms, comorbid pain, other nonspecific somatic symptoms, and comorbid psychiatric conditions diagnosed using DSM-IV-TR criteria were common and are documented in Table 3.

**Table 3:** Comorbid neurological, medical and psychiatric diagnoses and comorbid non-specific somatic symptoms

	*n*	%
Current comorbid neurological condition		
Epileptic seizures (one was part of a congenital syndrome, see below)	7	11.67
Congenital condition with neurological manifestations (neurofibromatosis Type 1 with hydrocephalus, epilepsy, and ocular gliomas; chromosome deletion 8 with spontaneous intraventricular bleeds, hydrocephalus, and ventriculo-peritoneal shunting procedures)	2	3.33
Left cerebral atrophy of unknown cause (unchanging over time)	1	1.67
Cerebral palsy	1	1.67
Hereditary angioedema	1	1.67
Tuberous sclerosis	1	1.67
Cerebellopontine angle cavernoma	1	1.67
Migraine (one child’s migraines were accompanied by hemiplegia)	2	3.33
Developmental delay	2	3.33
Past history of a neurological insult to the central nervous system		
Past history of viral meningitis	2	3.33
Past history of chemotherapy	1	1.67
Other conditions or vulnerabilities		
Type 1 diabetes	1	1.67
Past history of Bell’s palsy	1	1.67
Hypermobility	4	6.67
Fainting secondary to orthostatic stress (2 girls, 1 boy)	3	5
Postural tachycardia syndrome (POTTS)	5	8.33
Borderline IQ	8	13.33
Comorbid psychological conditions and somatic symptoms		
Motor-sensory conversion symptoms	32	53.33
Anxiety disorder (excluding PTSD and panic disorder)	22	36.67
PTSD	7	11.67
Panic disorder	7	11.67
Depression	10	16.67
Dissociative symptoms (loss of memory or capacity to recognize family members)	18	30
Behavioral disorder	3	5
Eating disorder	1	1.67
Comorbid pain	41	68.33
Disturbed sleep	23	38.33
Any nonspecific somatic symptom (excluding pain)	53	88.33
Dizziness	40	66.67
Breathlessness	33	55
Nausea	25	41.67
Fatigue	25	41.67
Heart pounding	20	33.33
Pins and needles	11	18.33

### Control group

The control group consisted of 50 children/adolescents—27 girls and 23 boys, aged 7–17 years (mean = 13.63; SD = 2.50). Seventeen children were from control group 1 (healthy controls), 25 from control group 2 (inpatient psychiatry controls: 8 with other functional neurological symptoms; 6 with somatoform pain; and 11 with depression, self-harm, or behavioral symptoms), and 8 from control group 3 (epilepsy controls). There were no significant differences between patients and controls regarding age [*t*(108) = −0.183; *p* = .855] and sex (*χ* ^2^ = 2.99; *p* = .08).

### Missing data

Baseline respiratory rates recorded at the beginning of the MyCalmBeat assessment were available for 52/60 children/adolescents with PNES and for 28/50 controls. HV-challenge data were available for all participants but were incomplete for 14 children/adolescents with PNES—missing the end part of the recovery period—because the procedure had been terminated early.

### Baseline respiratory rates at MyCalmBeat assessment

Patients with PNES had higher baseline respiratory rates than controls [patients: mean = 24.62; SD = 8.58; median = 24.50; range 8–50 bpm vs. controls: mean = 19.71; SD = 4.62; median = 20; range 8–31 bpm (*t*(77.995) = 3.323, *p* = .001)] (see Table 4). Using clinical reference ranges, an elevated baseline respiratory rate (above the 75th percentile) was documented in 36/52 (69%) patients with PNES (20–50 breaths per minute; mean = 28.61; SD = 7.121; median = 26.50) and 11/28 (39%) controls (21–31 breaths per minute; mean = 23.73; SD = 2.901; median = 23.00) (*χ* ^2^ = 6.7343; *df* = 1; *p* = .009).

**Table 4:** Between-group differences in respiratory and heart measures during the hyperventilation challenge

Measure	PNES group mean value (*n* = 60)	Control group mean value (*n* = 50)	*t*	*p*	Cohen’s d effect size
Baseline HR and respiratory rate					
Baseline HR (bpm)^a^	90.97	77.24 (*n* = 17, healthy controls)	4.008	<.001	0.96
Clinical baseline breathing rate (breaths per minute)	24.62 (*n* = 52 due to missing data)	19.71 (*n* = 28 due to missing data)	3.323	.001	0.64
Hyperventilation challenge PCO_2_ measures					
Baseline PCO_2_ (mmHg)	36.97	41.14	−5.53	<.001	1.07
∆PCO_2_ (mmHg)	13.14	14.92	−2.07	.041	0.40
Trough-PCO_2_ (mmHg)	23.85	26.36	−3.09	.003	0.59
Time to trough PCO_2_ (min)	5.13	5.24	.552	.582	0.11
Rate of PCO_2_ drop (mmHg/min)	2.65	2.98	−1.65	.102	0.32
Recovery measures					
	*n* = 51	*n* = 48			
Rate of PCO_2_ recovery (mmHg/min)	1.94	3.18	−4.09	<.001	0.91
	*n* = 47	*n* = 50			
Final PCO_2_ (mmHg)	36.21	42.36	−6.37	<.001	1.40
Other					

^a^Analyses with HR were done between the PNES group and healthy controls because the other clinical groups also showed elevated baseline HR (see Table 3)

### Baseline HR at hyperventilation-challenge assessment

In line with findings from a previous study [10], patients with PNES had higher baseline HR than healthy controls (patients: mean = 90.97; SD = 12.78; median = 91.00; range 64–127 bpm vs. healthy controls: mean = 77.24; SD = 11.23; median = 79.00; range 55–97 bpm [*t*(75) = 4.008, *p* < .001)] (see Table 4). Using clinical reference ranges, an elevated baseline HR (above the 75th percentile) was documented in 29/60 (48%) patients with PNES and 1/17 (6%) healthy controls) (*χ* ^2^ = 10.04; *df* = 1; *p* = .002).

### Relationship between respiration rate and HR

Increases in respiratory rate after the instruction to hyperventilate were followed by an increase in HR in both patients (mean change = 21 bpm) and controls (mean change = 23 bpm) [*t*(108) = −0.79, *p* = .43].

### Arterial PCO_2_ during the HV challenge of the vEEG test

**Fig. 1:**
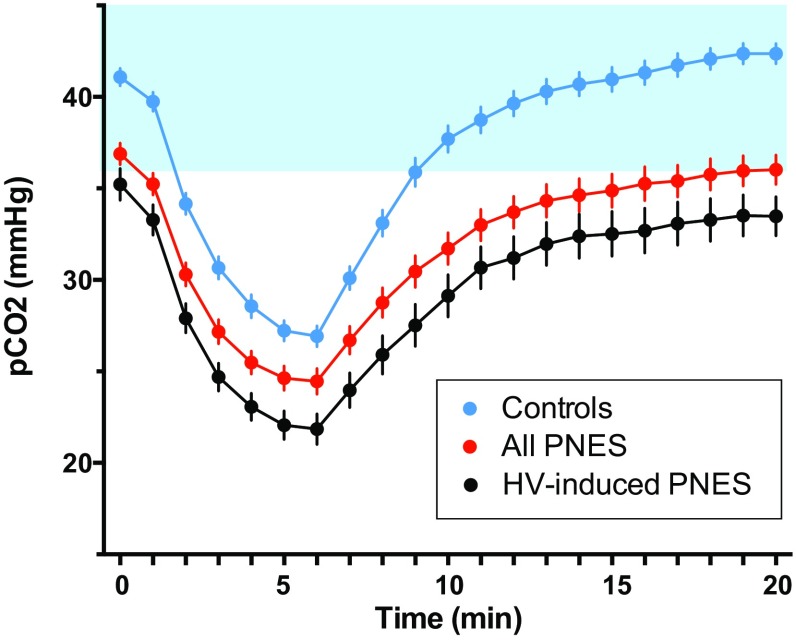
Hyperventilation profiles in children and adolescents assessed for PNES and in controls. The *shaded blue area* depicts the homeostatic range for arterial CO2. The *top blue line* depicts controls. Controls showed a clear pattern of PCO_2_ changes during the HV task: a baseline PCO_2_ within the homeostatic range, a steep drop in PCO_2_ during HV, and a prompt return to homeostasis during recovery. The *middle red line* depicts the 60 children and adolescents with PNES who participated in the study. Children and adolescents with PNES showed a downwardly skewed HV-challenge profile suggesting difficulties with PCO_2_ regulation. The *bottom black line* depicts the subgroup of 32 children and adolescents who whose PNES were typically preceded by—“triggered by”—HV

The PNES group (in red) and control group (in blue) (Fig. 1) showed clear differences in the PCO_2_ response to the HV challenge [*F*(1, 94) = 34.26; *p* < .001; partial eta square = 0.267]. The HV-challenge profile of the PNES group was shifted downward (away from homeostatic range) and characterized by a significantly lower baseline PCO_2_, lower trough PCO_2_, slower rate of PCO_2_ recovery, and lower final PCO_2_. Whereas the time to trough PCO_2_ and the rate of PCO_2_ drop were the same as in controls, the actual ∆PCO_2_ with hyperventilation was smaller (see Table 4). Further analysis showed that 28/60 PNES patients (47%) showed a skewed HV-challenge profile—defined as a baseline PCO_2_ < 36 mmHg, trough PCO_2_ ≤ 20 mmHg, or failure of recovery back to homeostasis—compared to only 4/50 (8%) controls (*χ* ^2^ = 19.77; *df* = 1; *p* < .001) (see Table 5). During the HV challenge, symptoms of cerebral hypoxia—dizziness, head dropping, staring, visual changes, or changes in consciousness—were almost five times more frequent in patients with PNES (23/60) than in controls (4/50) (*χ* ^2^ = 13.55; *df* = 1; *p* < .001).

**Table 5:** Abnormal HV profiles in participants with PNES vs. controls

Categorical variable	PNES group *n*	Control group *n*	Chi squared (*χ* ^2^)	*p* value
Skewed HV profile (skew in any of the three components below)	26/60	4/50	19.77	<.001
Hypocapnia at baseline (PCO_2_ < 36 mmHg)	21/60	3/50	13.45	<.001
Lowest PCO_2_ ≤ 20 mmHg	16/60	2/50	10.24	.001
Failure of recovery to homeostasis, or final PCO_2_ <36 mmHg	19/47	1/50	21.86	<.001

Observation of PNES episodes during treatment admissions showed that in 32 of the 60 patients, paroxysmal increases in ventilation—probable HV—occurred immediately prior to PNES episodes. The PCO_2_ response to HV challenge of this “HV-induced PNES” subgroup is shown in black on Fig. 1. The average profile of this subgroup of patients was shifted even more downward (away from homeostatic range) than in the PNES group as a whole.

## Discussion

The PNES Hyperventilation Study used respiratory measures—baseline respiratory rate at clinical assessment and percutaneous CO_2_ monitoring during the hyperventilation component of routine vEEG—to assess motor readiness and CO_2_ regulation in children/adolescents with PNES and in controls. Baseline HR was also documented. As a group, patients with PNES had elevated baseline respiratory rates, elevated baseline HR, and skewed HV-challenge profiles, with significantly lower levels of arterial CO_2_ at all timepoints of the HV challenge. Symptoms of cerebral hypoxia were also almost five times more frequent in patients with PNES than in controls during the HV challenge. The study findings suggest patients presented in a state of readiness-for-action characterized by high arousal coupled with activation of the respiratory motor system and increases in ventilation. Their CO_2_ profiles during the HV challenge were shifted downward away from the homeostatic range, suggesting that excessive activation of the respiratory motor system, coupled with difficulties in regulating CO_2_, may contribute to the functional neural mechanisms that underpin PNES.

Although motor activity (mediated by the skeletomotor system) and sympathetic arousal (mediated by the visceromotor system) involve separate neural systems, they are interrelated and activated in a coordinated manner. Activation of the motor system (including its ventilatory component) is always coupled with sympathetic arousal, and sympathetic arousal is always coupled with activation of the motor system (at least an increase in muscle tone) [54–56]. This close interconnection between motor activation, including ventilation, and sympathetic arousal is a product of brain structure and function; the cortical areas involved in preparing and generating motor (skeletomotor) outputs overlap with those from which sympathetic (visceromotor) outputs originate [56]. Motor activity, including states of motor readiness and motor preparation, are accompanied by concurrent changes in ventilation and sympathetic output that match the metabolic demands of the movement. In the face of perceived threat, increased ventilation and a correlative increase in sympathetic arousal are part of the body’s preparation for action [54, 55]. In our study, however, we found that children/adolescents with PNES had low baseline PCO_2_ and failure of PCO_2_ recovery back to homeostasis following the HV challenge. These findings indicate overreactivity and dysregulation of the finely tuned interconnections between motor activity and sympathetic arousal. This result matches, in part, the results of our previous studies, in which all children/adolescents with functional neurological symptoms—including the PNES subgroup—showed increased arousal and motor readiness to emotion stimuli. A striking difference in the PNES subgroup, however, is their excessive activation of the respiratory motor system and the associated dysregulation of CO_2_ control, which may well contribute to their propensity for PNES.

As noted in the introduction, neurophysiological studies show that patients with PNES demonstrate altered properties in brain networks—changes (both decreases and increases) in the relationship between cortical brain regions that normally function together [5, 16–19, 57–59]—resulting in functional disruptions in both the brain’s horizontal integration (between cortical regions) and its vertical integration (between cortical and subcortical regions). These functional disruptions are likely to weaken executive control over motor areas and to prioritize reflexive control of behaviors, thereby enabling release or activation of prewired autonomous motor programs [4]—presumably those that are represented at the level of the basal ganglia, midbrain, and brainstem.

We suggest that in children/adolescents, HV may contribute to functional brain disruptions and, via two different mechanisms, to a propensity to PNES. First, because of the close coupling between respiration and sympathetic arousal, HV-induced increases in cortical arousal could trigger PNES by disrupting cortical executive control over the motor cortex and cortico–basal ganglia–thalamocortical loops. Since children/adolescents with PNES show increased baseline autonomic and cortical arousal [10, 13, 15], even minor elevations in arousal mediated by HV could disrupt cortical control and serve to disrupt cortical integration [60–62] and trigger PNES. Second, if HV continues beyond the excitatory stage into the hypoxic stage, then hypoxia-related impairments in the cortex and basal ganglia [63] will likewise contribute to dysfunction in top–down control mechanisms within the motor system and to disruptions in the brain’s vertical integration. Because the basal ganglia and brain stem mediate changes in muscle tone and consciousness [64], a subset of PNES patients with HV-induced hypoxia will experience changes in consciousness and losses of muscle tone.

Contrary to our original hypothesis, we found no significant differences between patients with PNES and controls in time to lowest PCO_2_ levels and in rates of PCO_2_ drop, and the PNES group had a smaller, not greater, ∆PCO_2_. These findings are consistent with a downwardly skewed HV-challenge profile, where the ∆PCO_2_ was smaller because patients had decreased reserves and reached a floor effect, as evidenced by central symptoms of HV-induced hypoxia. Son et al. [65] likewise found that ∆PCO_2_ was not useful in the assessment of EEG slowing (and of the associated cerebral dysfunction) in child/adolescent patients.

All the PNES patients in this study reported stressors prior to the onset of PNES—illness, injury, emotional distress secondary to adverse life events, or psychological trauma. Not all stressors involved psychological distress per se. These findings highlight that from the perspective of the body, the types of stressors that effect brain-body stress systems and that predispose children/adolescents to PNES go well beyond those that are subsumed under the umbrella of “psychogenic”. From the perspective of the body, various stressors—pain, inflammation, and disruptions of the circadian cycle, as well as psychological stress and psychological trauma—may function to activate a sensitized, highly reactive stress system and to change functional organization of the brain [43, 60, 61, 66–70], with PNES being an unwanted by-product of the body’s response to threat.

This study was carried out as part of our busy routine clinical practice. It has a number of limitations. First, although transcutaneous measures of PCO_2_ correlate well with arterial blood levels, the diffusion of CO_2_ from the blood to the skin is slow; changes in arterial PCO_2_ are registered with a delay of 1–2 min, and sharp changes are blunted [71]. Second, skewed HV-challenge profiles—and trough-PCO_2_ levels, in particular—in combination with EEG slowing are an approximate measure of HV-induced neurophysiological changes. In future studies, more direct measures of vasoconstriction/cerebral artery blood flow (via Doppler ultrasound) would help to clarify underlying neurophysiological mechanisms, as would measuring changes in patterns of brain oxygenation (via functional magnetic resonance imaging). Third, no simple, direct measures of cortical arousal are currently available. Techniques to measure such changes on a second-to-second basis would help to clarify underlying neurophysiological mechanisms. Fourth, respiratory rates were measured at clinical assessment but not during the vEEG procedure. That said, arterial CO_2_ is the gold standard for measuring HV. Elevated respiratory rates alone (without consideration of tidal volume) can flag the possibility of HV but do not measure it directly. Finally, it would have been useful to collect data about the contribution of disturbed sleep and the menstrual cycle (in 27/60 postpubertal girls) to the upregulation of the stress system [72, 73].

Despite limitations, this study contributes to current research efforts to identify the biological mechanisms that underpin PNES. The study has important implications for assessing and treating PNES in paediatric practice. Children/adolescents with PNES present in a state of increased baseline arousal. In a subset, increased arousal is coupled with excessive activation and reactivity of the motor respiratory system, and in these children/adolescents, PNES can be preceded and triggered by paroxysmal increases in arousal coupled with HV. Identifying patients in whom HV appears to trigger PNES is important because the majority of child/adolescent patients are able to utilize breathing interventions to slow their breathing, downregulate arousal, and avert PNES episodes. Once the PNES have come under control, the children, adolescents, and families are better placed to address the psychological or physiological threats that triggered their PNES in the first place.

## Electronic supplementary material

Below is the link to the electronic supplementary material.

Supplementary material 1 (DOCX 51 kb)

## Footnotes

1 In the earlier studies by Kozlowska et al., children/adolescents with PNES made up 51% (*n* = 29/57) of the samples [10–12]. In the most recent study of cortical arousal, children/adolescents with PNES made up 56% (*n* = 32/57) of the sample [13].
