# Supplementary material for: The respiratory control of carbon dioxide in children and adolescents referred for treatment of psychogenic non-epileptic seizures
Source: Eur Child Adolesc Psychiatry. 2017 Mar 24;26(10):1207–17. doi: 10.1007/s00787-017-0976-0 (PMC5610228; doi:10.1007/s00787-017-0976-0)
Supplement: Supplementary file 1 — Supplementary material 1 (DOCX 51 kb) [file 787_2017_976_MOESM1_ESM.docx]

**Supplementary on-line materials**

**Supplemental Text Box 1: The neurophysiology of Hyperventilation**

| Hyperventilation, breathing in excess of metabolic demands, causes a broad range of neurophysiological changes both in the central and in the peripheral nervous systems.  ***Central changes***  **Phase 1:** Hyperventilation involves an initial excitatory phase, with an increase in cortical excitability, that is followed in some individuals by a hypoxic phase, with a decrease in cortical function. Excitatory changes include increased cell membrane permeability, increased metabolism and oxygen consumption, hypopolization of neurones, increased cortical excitability in widely distributed networks (including motor and visual cortex) [1-4], and decreases in intracortical inhibition [1]. These initial changes that occur with hyperventilation are similar to those seen with sympathetic nervous system stimulation and mild-moderate activation of cortical arousal systems.  **Phase 2:** In some individuals, hyperventilation may continue, producing an increase in cerebral hypoxia and consequent decrease in cerebral function. The relevant cascade of changes include the following: lowered arterial CO2 and an increase in arterial pH (alkalosis) [5,6]; cerebral artery vasoconstriction [7-9] and decreased blood flow [10-13]; increased binding of oxygen to haemoglobin (Bohr effect) [14]; decreased brain tissue oxygenation and cerebral oxygen metabolism [15,16]; and increased glycolysis with production of lactic acid by neurons [17]. The cerebral cortex and basal ganglia—which mediate changes in consciousness—are the brain regions most sensitive to hypoxia, followed by the hypothalamus and midbrain, with the medulla and pons being most resistant, leaving their programs available for activation even in states of hypoxia [18]. With HV, and in comparison to adults, children and adolescents show more pronounced decreases in cerebral blood flow [12,10] and more pronounced hypoxia-related EEG slowing [19-21]. The latter is associated with changes in cognitive processing and with altered awareness and responsiveness including states of presyncope or syncope [22-26]. In healthy adults, central symptoms are more likely if PCO2 falls to 20mmHG (SD, 3–4mmHG; range, 14–29mmHG)[27].  ***Peripheral changes***  Peripheral neurological effects of HV include hypopolarization of neurons, increased excitability of sensory and motor axons in the peripheral nervous system, which can cause increased muscle excitability, paraesthesias, and carpo-pedal spasms [28]. Cardiac effects include increased contractility and increased oxygen extraction [29], and vasoconstriction of coronary arteries induce HV-induced chest pain (see Kozlowska 2013 for review) [30]. When HV continues without inducing significant hypoxia, some individuals experience paraesthesias and carpo-pedal spasms (anaesthesia and tetany seem not to develop once hypoxia sets in) [6,24]. |
| --- |

**REFERENCES FOR SUPPLEMENTARY TEXT BOX 1**

1. Sparing R, Dafotakis M, Buelte D, Meister IG, Noth J (2007) Excitability of human motor and visual cortex before, during, and after hyperventilation. J Appl Physiol (1985) 102 (1):406-411. doi:10.1152/japplphysiol.00770.2006

2. Jensen O, Hari R, Kaila K (2002) Visually evoked gamma responses in the human brain are enhanced during voluntary hyperventilation. NeuroImage 15 (3):575-586. doi:10.1006/nimg.2001.1013

3. Carbon M, Wübbeler G, Trahms L, Curio G (2000) Hyperventilation-induced human cerebral magnetic fields noninvasively monitored by multichannel `direct current' magnetoencephalography. Neuroscience letters 287:227-230

4. Stenkamp K, Palva JM, Uusisaari M, Schuchmann S, Schmitz D, Heinemann U, Kaila K (2001) Enhanced temporal stability of cholinergic hippocampal gamma oscillations following respiratory alkalosis in vitro. Journal of neurophysiology 85 (5):2063-2069

5. Blinn KA, Noell WK (1949) Continuous measurement of alveolar CO2 tension during the hyperventilation test in routine electroencephalography. Electroencephalography and clinical neurophysiology 1 (3):333-342

6. Engel GL, Ferris EB, Logan M (1947) Hyperventilation: Analysis of clinical symptomatology. . Annals of internal medicine 27:683-704

7. Gibbs EL, Gibbs FA, Lennox WG, Nims LF (1942) Regulation of cerebral carbon dioxide. Archives of Neurology and Psychiatry 47:879

8. Hauge A, Thoresen M, Walloe L (1980) Changes in cerebral blood flow during hyperventilation and CO2-breathing measured transcutaneously in humans by a bidirectional, pulsed, ultrasound Doppler blood velocitymeter. Acta physiologica Scandinavica 110 (2):167-173

9. Kraaier V, van Huffelen AC, Wieneke GH (1988) Changes in quantitative EEG and blood flow velocity due to standardized hyperventilation; a model of transient ischaemia in young human subjects. Electroencephalography and clinical neurophysiology 70 (5):377-387

10. Yamaguchi F, Meyer JS, Sakai F, Yamamoto M (1979) Normal human aging and cerebral vasoconstrictive responses to hypocapnia. Journal of the neurological sciences 44 (1):87-94

11. Gibbs DM (1992) Hyperventilation-induced cerebral ischemia in panic disorder and effect of nimodipine. American Journal of Psychiatry 149 (11):1589-1591

12. Yamatani M, Konishi T, Murakami M, Okuda T (1994) Hyperventilation activation on EEG recording in childhood. Epilepsia 35 (6):1199-1203

13. Ball S, Shekhar A (1997) Basilar artery response to hyperventilation in panic disorder. American Journal of Psychiatry 154 (11):1603-1604

14. Bohr C, Hasselbalch K, Krogh A (1904) Über einen in biologischer Beziehung wichtigen Einfluss, den die Kohlensäurespannung

des Blutes auf dessen Sauerstoffbindung übt [Concerning a Biologically Important Relationship -

The Influence of the Carbon Dioxide Content of Blood on its Oxygen Binding]. Skandin Arch Physiol 16:401-412

15. Yang R, Brugniaux J, Dhaliwal H, Beaudin AE, Eliasziw M, Poulin MJ, Dunn JF (2015) Studying cerebral hemodynamics and metabolism using simultaneous near-infrared spectroscopy and transcranial Doppler ultrasound: a hyperventilation and caffeine study. Physiological reports 3 (4). doi:10.14814/phy2.12378

16. Meng L, Mantulin WW, Alexander BS, Cerussi AE, Tromberg BJ, Yu Z, Laning K, Kain ZN, Cannesson M, Gelb AW (2012) Head-up tilt and hyperventilation produce similar changes in cerebral oxygenation and blood volume: an observational comparison study using frequency-domain near-infrared spectroscopy. Canadian journal of anaesthesia = Journal canadien d'anesthesie 59 (4):357-365. doi:10.1007/s12630-011-9662-8

17. Siesjo BK, Kjallquist A (1969) A new theory for the regulation of the extracellular pH in the brain. Scandinavian journal of clinical and laboratory investigation 24 (1):1-9

18. Gastaut H (1974) Syncopes: Generalised anoxic cerebral seizures. In: Magnus O, Lorentz de Hoos AM (eds) The Epilepsies. Handbook of Clinical Neurology, vol 15. New Holland, Amsterdam, pp 815-835

19. Gotoh F, Meyer JS, Takagi Y (1965) Cerebral Effects of Hyperventilation in Man. Archives of neurology 12:410-423

20. Gibbs FA, Gibbs EL, Lennox WG (1943) Electroencephalographic response to overventilation and its relations to age. Journal of Pediatrics 23:497-505

21. Son S, Kwon OY, Jung S, Kim YS, Kim SK, Kang H, Park KJ, Choi NC, Lim BH (2012) Relationship between Hyperventilation-Induced Electroencephalographic Changes and PCO2 Level. Journal of epilepsy research 2 (1):5-9. doi:10.14581/jer.12002

22. Okel BB, Hurst JW (1961) Prolonged hyperventilation in man. Associated electrolyte changes and subjective symptoms. Archives of internal medicine 108:757-762

23. Allen TE, Agus B (1968) Hyperventilation leading to hallucinations. The American journal of psychiatry 125 (5):632-637

24. North KN, Ouvrier RA, Nugent M (1990) Pseudoseizures caused by hyperventilation resembling absence epilepsy. J Child Neurol 5 (4):288-294

25. Epstein MA, Duchowny M, Jayakar P, Resnick TJ, Alvarez LA (1994) Altered responsiveness during hyperventilation-induced EEG slowing: a non-epileptic phenomenon in normal children. Epilepsia 35 (6):1204-1207

26. Barker A, Ng J, Rittey CD, Kandler RH, Mordekar SR (2012) Outcome of children with hyperventilation-induced high-amplitude rhythmic slow activity with altered awareness. Developmental medicine and child neurology 54 (11):1001-1005. doi:10.1111/j.1469-8749.2012.04337.x

27. Rafferty GF, Saisch SG, Gardner WN (1992) Relation of hypocapnic symptoms to rate of fall of end-tidal PCO2 in normal subjects. Respiratory medicine 86 (4):335-340

28. Macefield G, Burke D (1991) Paraesthesiae and tetany induced by voluntary hyperventilation. Increased excitability of human cutaneous and motor axons. Brain : a journal of neurology 114 ( Pt 1B):527-540

29. Laffey JG, Kavanagh BP (2002) Hypocapnia. The New England journal of medicine 347 (1):43-53. doi:10.1056/NEJMra012457

30. Kozlowska K (2013) Stress, Distress, and Bodytalk: Co-constructing Formulations with Patients Who Present with Somatic Symptoms. Harvard review of psychiatry 21 (6):314-333. doi:10.1097/HRP.0000000000000008
